# Successful Percutaneous Coronary Intervention Using Intravascular Ultrasound-Guided Rewiring Technique in a Case of Spontaneous Coronary Artery Dissection Involving Left Main Bifurcation

**DOI:** 10.1155/2020/8890538

**Published:** 2020-07-02

**Authors:** Yohei Numasawa, Souichi Yokokura, Yasuhiro Hitomi, Shohei Imaeda, Makoto Tanaka, Ryota Tabei, Masaki Kodaira

**Affiliations:** Department of Cardiology, Japanese Red Cross Ashikaga Hospital, Ashikaga, Japan

## Abstract

We herein describe a 49-year-old woman without significant cardiovascular risk factors who was transferred to our hospital with sudden onset of chest pain. The patient was diagnosed with non-ST-elevation acute myocardial infarction, and coronary angiography revealed a dissection at the proximal site of the left anterior descending artery (LAD) extending from the left main trunk (LMT) suggestive of spontaneous coronary artery dissection (SCAD). Because coronary flow was impaired after contrast injection and the patient had chest pain with ST elevation, urgent percutaneous coronary intervention was performed. The first guide wire was initially introduced from the LMT to the distal LAD, but intravascular ultrasound (IVUS) imaging revealed that the guide wire had passed through the true lumen of the left coronary artery ostium, false lumen at the ostium of the left circumflex artery, and true lumen of the distal LAD. We then reinserted another guide wire using an IVUS-guided rewiring technique from the true lumen of the LMT to the distal LAD. Finally, a drug-eluting stent was deployed to cover the dissected segment, and final coronary angiography revealed acceptable results with a patent left circumflex artery. This case report highlights that physicians should consider SCAD among the differential diagnoses in patients presenting with acute coronary syndrome, particularly in young women. In the present case, IVUS played a pivotal role in not only detecting the arterial dissection but also correctly introducing the guide wire into the true lumen.

## 1. Introduction

Spontaneous coronary artery dissection (SCAD) is a relatively rare but important cause of acute coronary syndrome, especially in young women without significant cardiovascular risk factors and coronary atherosclerosis [1, 2]. In general, conservative medical therapy is recommended in clinically stable patients with SCAD because observational studies have shown favorable short-term outcomes and a high rate of spontaneous angiographic healing of the dissected segment [2–4]. In contrast, in patients with high-risk features such as ongoing ischemia with ST elevation, dissection involving the left main trunk (LMT), and hemodynamic instability, urgent revascularization strategies including percutaneous coronary intervention (PCI) or coronary artery bypass surgery should be considered for the treatment of SCAD [5–7]. However, PCI has a relatively low success rate and a high complication rate in patients with SCAD, and a previous study showed that the failure rate of PCI was 53% [8].

We herein describe a 49-year-old woman who was diagnosed with non-ST-elevation acute myocardial infarction due to SCAD involving left main bifurcation. Because the coronary blood flow of the left anterior descending artery (LAD) was impaired by an enlargement of the false lumen after contrast injection, we performed urgent PCI. The first guide wire was initially introduced from the LMT to the distal LAD, but intravascular ultrasound (IVUS) imaging revealed that the guide wire had passed through the false lumen at the ostium of the left circumflex artery (LCX). We then successfully reinserted another guide wire using an IVUS-guided rewiring technique from the true lumen of the LMT to the distal LAD. In this case, IVUS played a pivotal role in not only detecting the dissection but also introducing the guide wire into the compressed true lumen.

## 2. Case Presentation

A 49-year-old woman without significant cardiovascular risk factors was transferred to our hospital with a 1-hour history of sudden-onset chest pain. She had taken no medication and experienced no physical or emotional stress preceding the chest pain. On arrival, physical examination revealed stable vital signs with no abnormal findings. Electrocardiography revealed no clear findings of ischemic ST-T changes or prior *Q*-wave myocardial infarction, but laboratory testing showed elevated cardiac enzymes such as troponin T (0.855 ng/mL) as well as creatine phosphokinase (565 U/L) and its MB isozyme (66 U/L). Bedside transthoracic echocardiography revealed impaired contractility in the focal apical segment of the left ventricle, but there was no clear finding of basal hypercontractility or an apical ballooning sign suggestive of stress-induced cardiomyopathy. Overall, the estimated ejection fraction of the left ventricle was broadly preserved (60%). The patient was diagnosed with non-ST-elevation acute myocardial infarction. Because her condition was stable without chest pain on arrival, we initially administered conservative medical therapy including aspirin, prasugrel, nicorandil, perindopril, pitavastatin, and bisoprolol.

**Figure 1:**
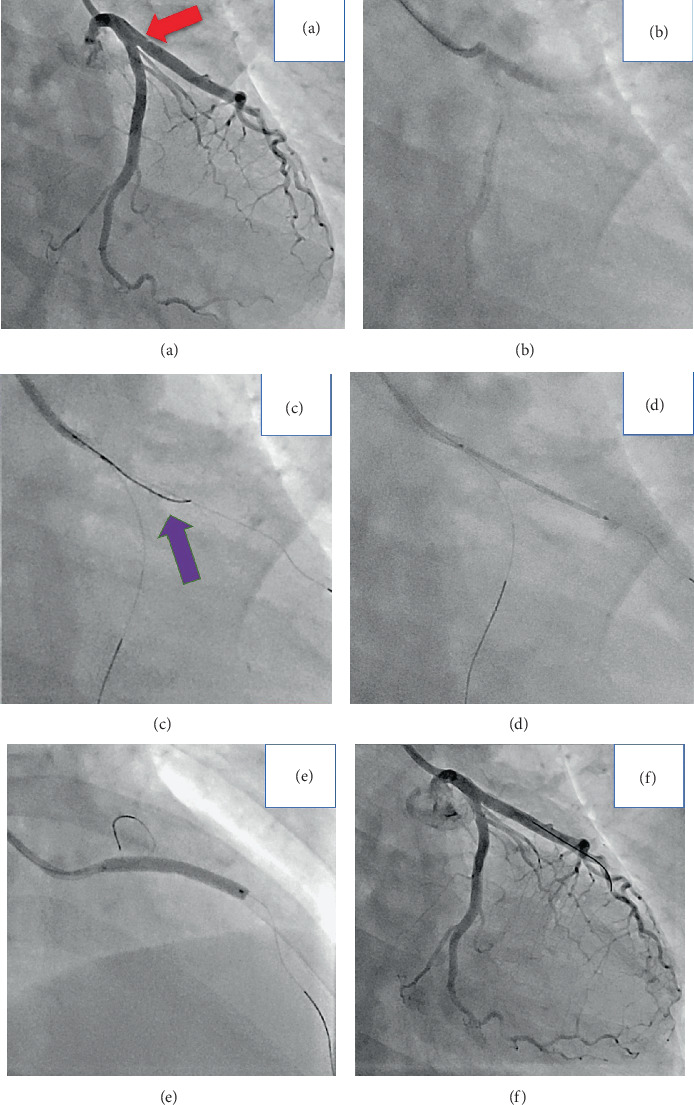
Coronary angiography and percutaneous coronary intervention. (a) Left coronary angiography (right anterior oblique caudal view) revealed a dissection at the proximal site of the LAD extending from the LMT, suggestive of type 1 spontaneous coronary artery dissection (red arrow). (b) Coronary blood flow of the LAD was impaired after coronary angiography. (c) The IVUS-guided rewiring technique was performed, and the third guide wire (purple arrow) was inserted from the true lumen of the LCX to the true lumen of the LAD by referring to the IVUS findings of the dissected segment. (d and e) A drug-eluting stent was deployed to cover the dissected segment. (f) Final coronary angiography revealed acceptable results. LMT: left main trunk; LAD: left anterior descending artery; LCX: left circumflex artery; IVUS: intravascular ultrasound.

**Figure 2:**
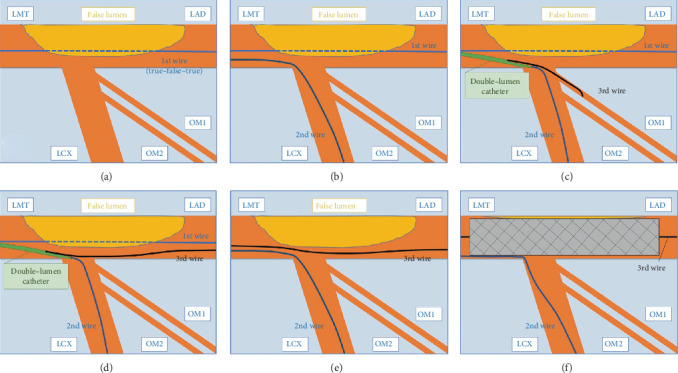
The scheme of the percutaneous coronary intervention. (a) The first guide wire passed through the false lumen from the midportion of the LMT to the proximal site of the LAD. (b) The second guide wire was inserted into the true lumen of the LCX. (c) The third guide wire was initially introduced into the true lumen of the obtuse marginal branch using a double-lumen catheter that was placed in the true lumen of the LCX. (d) We pulled the third guide wire back and controlled it toward the true lumen of the LAD, which was located under the first guide wire from the right anterior oblique and caudal view. (e) After the successful rewiring, the first guide wire was removed. (f) A drug-eluting stent was deployed from the LMT to LAD. LMT: left main trunk; LAD: left anterior descending artery; LCX: left circumflex artery; OM: obtuse marginal branch.

**Figure 3:**
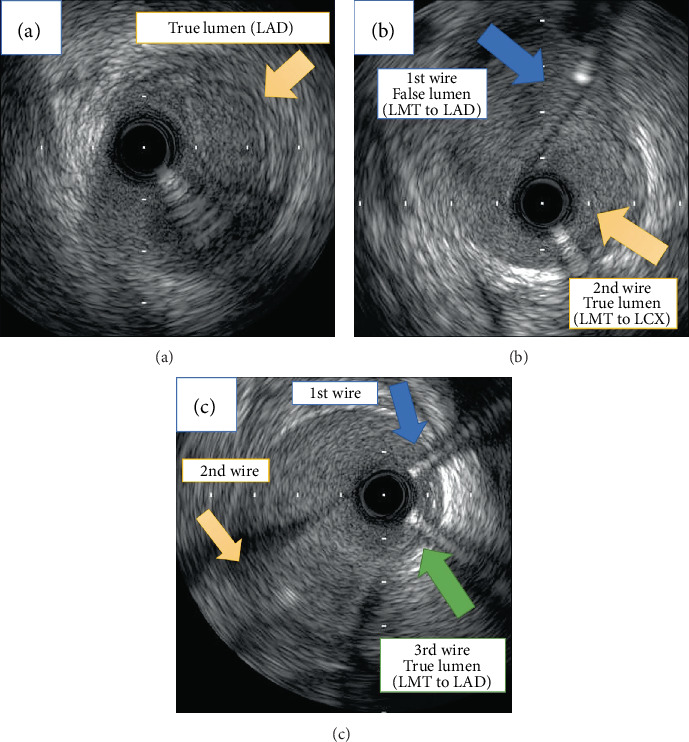
IVUS findings during percutaneous coronary intervention. (a) The first guide wire passed through the false lumen from the midportion of the LMT to the proximal site of the LAD, and the true lumen of the LAD was compressed by the enlarged false lumen (orange arrow). (b) The second guide wire was inserted into the LCX, and IVUS imaging confirmed that it had been placed in the true lumen from the LMT to LCX (yellow arrow). (c) The third guide wire passed entirely through the true lumen from the LMT to LAD (green arrow). LMT: left main trunk; LAD: left anterior descending artery; LCX: left circumflex artery; IVUS: intravascular ultrasound.

Three days later, we electively performed coronary angiography via the right radial artery using a 6 Fr sheath to determine the etiology of the myocardial injury. Coronary angiography showed no significant atherosclerotic stenosis in the right coronary artery and LCX, but a dissection was found from the midportion of the LMT to the proximal site of the LAD, suggestive of type 1 SCAD (Figure 1(a), Video1) [9]. Because the coronary blood flow of the LAD was impaired from thrombolysis in myocardial infarction (TIMI) flow grade III to I after contrast injection (Figure 1(b)) and the patient had chest pain with ST elevation, we decided to perform urgent PCI. The first wire (SION Blue; Asahi Intecc Co., Ltd., Nagoya, Japan) was initially introduced into the distal LAD, but IVUS imaging revealed that the guide wire had passed through the true lumen of the left coronary artery ostium, false lumen at the ostium of the LCX, and true lumen of the distal LAD (true-false-true) (Figure 2(a)). In addition, the true lumen of the proximal LAD was compressed by the false lumen (Figure 3(a)), and no significant atherosclerosis was observed within the vessel on IVUS. We then inserted the second guide wire (SION Blue; Asahi Intecc Co., Ltd.) into the LCX (Figure 2(b)), and IVUS imaging confirmed that it had been placed in the true lumen from the LMT to LCX (Figure 3(b)). Next, we inserted the third guide wire (SION Blue; Asahi Intecc Co., Ltd.) from the true lumen of the LCX to the compressed true lumen of the LAD using a double-lumen catheter (Crusade Type R; Kaneka Corporation, Tokyo, Japan) by referring to the IVUS findings of the dissected segment (Video2). We initially inserted the third guide wire into the true lumen of the obtuse marginal branch using a double-lumen catheter that was placed in the true lumen of the LCX (Figure 2(c)). We then pulled the wire back and controlled it toward the true lumen of the LAD, which was located under the first guide wire from the right anterior oblique and caudal view (Figures 1(c) and 2(d)). The IVUS-guided rewiring technique was successful, and the subsequent IVUS imaging confirmed that the third guide wire had passed entirely through the true lumen from the LMT to LAD (Figure 3(c), Video3). After the successful rewiring from the true lumen of the LMT to distal LAD, we removed the first wire (Figure 2(e)) and deployed a drug-eluting stent (Resolute Onyx, 3.5 × 30 mm; Medtronic plc, Fridley, MN, USA) from the LMT to LAD (Figures 1(d), 1(e), and 2(f)). Because the blood flow of the LCX was preserved after stenting, we did not perform rewiring of the side branch and final kissing balloon inflation. Final coronary angiography showed acceptable results and TIMI grade III flow in both the LAD and LCX (Figure 1(f)).

**Figure 4:**
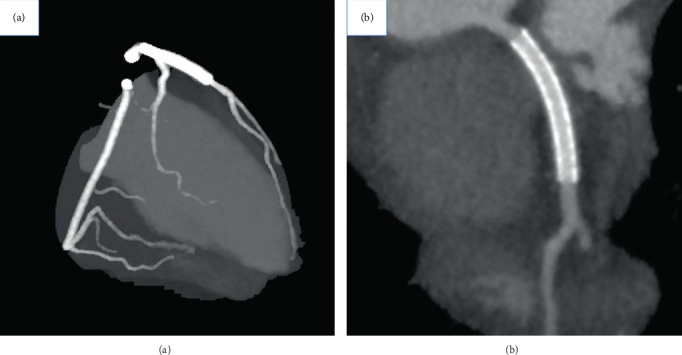
Coronary CT angiography performed 12 months after percutaneous coronary intervention. (a) Follow-up coronary CT angiography revealed no atherosclerotic stenosis in both the right and left coronary arteries. (b) There were no clear findings of in-stent restenosis or incomplete stent apposition. CT: computed tomography.

The postprocedural course was uneventful, and no major adverse cardiovascular events were observed. The peak levels of creatine phosphokinase and its MB isozyme were 1155 and 142 U/L, respectively. Follow-up coronary computed tomography (CT) angiography was performed 12 months after the procedure and showed acceptable results with no clear findings of in-stent restenosis or incomplete stent apposition (Figures 4(a) and 4(b)). The patients' clinical course was uneventful during an 18-month follow-up.

## 3. Discussion

This case report highlights that SCAD should always be included among the differential diagnoses in patients with acute coronary syndrome, particularly in young women. Furthermore, IVUS plays a pivotal role in detecting not only the presence of a dissection but also the optimal position of the guide wire in patients with SCAD.

The treatment strategy for SCAD is controversial because of the lack of randomized controlled clinical trials. Recent large-scale observational studies have suggested that conservative medical therapy is generally recommended in hemodynamically stable patients with SCAD because of the reportedly favorable short-term outcomes and high rate of spontaneous angiographic healing of the dissection [2–4]. However, urgent revascularization strategies should be considered in patients with SCAD complicated by high-risk features such as ongoing ischemia with TIMI flow grade 0 to I and/or ST-elevation findings on the electrocardiogram, dissection involving the LMT, and hemodynamic instability [5–7]. In addition, a previous study showed that a left main culprit was relatively common (13%) in patients with SCAD presenting with ST-elevation acute myocardial infarction [7]. In the present case, we decided to perform urgent PCI because the coronary flow was suddenly impaired from TIMI flow grade III to I (which was mainly due to the progression of the dissection involving the left main bifurcation after coronary angiography) and the patient had chest pain with ST elevation.

PCI for patients with SCAD is technically challenging mainly because of difficulties in wiring into the true lumen and the risk of extending the dissection or intramural hematoma by the catheter-based procedure [5, 6, 8]. Indeed, the reported rate of successful PCI in patients with SCAD ranges from 47% to 91% [5, 7, 8, 10]. In this case, it was difficult to insert the guide wire into the true lumen of the dissected segment involving the left main bifurcation. Although the first guide wire seemed to have been successfully introduced from the LMT to LAD, IVUS imaging confirmed that the wire had passed through the true lumen of the left coronary artery ostium, false lumen at the ostium of the LCX, and true lumen of the distal LAD (true-false-true). If we had deployed a drug-eluting stent from the LMT to LAD in this situation, the LCX would have been completely occluded. Thus, we inserted the second guide wire into the true lumen of the LCX and the third guide wire into the true lumen of the LMT and LAD using an IVUS-guided rewiring technique. Although the true lumen of the LAD was compressed by the enlarged false lumen, IVUS imaging from both the true lumen of the LCX and the false lumen of the LAD clearly confirmed that the true lumen of the proximal LAD was close to the ostium of the LCX and obtuse marginal branch. Therefore, we initially inserted the third guide wire into the true lumen of the obtuse marginal branch using a double-lumen catheter that was placed in the true lumen of the LCX; we then pulled the wire back and controlled it toward the true lumen of the LAD, which was located under the first guide wire from the right anterior oblique and caudal view. Although the IVUS-guided wiring technique is commonly used in patients undergoing PCI for chronic total occlusion lesions [11], the present case suggests that it is also useful during PCI for SCAD, especially that involving left main bifurcation. During the procedure, IVUS played a pivotal role in not only detecting the dissection but also introducing the guide wire into the compressed true lumen. After the successful rewiring into the true lumen, a drug-eluting stent was deployed to cover the dissected segment. Because the blood flow of the LCX was preserved after stenting and a complex procedure was considered to be associated with adverse outcomes, including extension of the dissection to the LCX, we did not perform rewiring of the side branch and final kissing balloon inflation.

Several PCI strategies have been reported in patients with SCAD, including balloon angioplasty using a cutting balloon, placement of stents, and use of bioabsorbable scaffolds [5, 6, 12]. Cutting balloon angioplasty to fenestrate the intima is one of the useful alternatives, especially in patients with SCAD characterized by a long and diffuse lesion [6, 13]. If decompression of the intramural hematoma occurs from the false lumen to the true lumen, stent implantation may be avoided in some cases [13, 14]. However, because the coronary arteries of patients with SCAD are vulnerable, cutting balloon angioplasty is associated with a risk of coronary perforation. In addition, creating a large flap after cutting balloon angioplasty, especially in the LMT, may increase the risk of fatal ischemia due to vessel occlusion. In the present case, because the dissection involved the left main bifurcation and could be covered by a single stent, we selected a direct stenting strategy using a drug-eluting stent.

In this case, because laboratory testing showed elevated cardiac enzymes on arrival and coronary angiography and IVUS imaging revealed no findings of atherosclerosis in the coronary arteries, the diagnosis of non-ST-elevation acute myocardial infarction due to SCAD was valid. However, catheter insertion and contrast injection might worsen the dissection and coronary blood flow of the LAD in such patients. Because iatrogenic catheter-induced coronary artery dissections are more common in patients with than without SCAD [15], we should have performed catheter manipulation and contrast injection more carefully. In addition, because the patient's condition was stable on admission and the culprit lesion was present in the proximal portion of the left coronary artery, coronary CT angiography prior to invasive coronary angiography might have been a noninvasive and reasonable alternative. Physicians should consider SCAD among the differential diagnoses in patients presenting with acute coronary syndrome; physicians should also be aware that both diagnostic coronary angiography and PCI are associated with a risk of iatrogenic catheter-induced coronary artery dissection in these patients. In this aspect, coronary CT angiography is a noninvasive and useful alternative in both the acute and chronic phases of SCAD in clinically stable patients [12, 16].

## 4. Conclusions

This case report suggests that IVUS is useful in not only detecting the dissection but also introducing the guide wire into the true lumen in patients with SCAD, especially that involving left main bifurcation.

## Data Availability

The data used to support the findings of this study are available from the corresponding author upon request.

## Abbreviations

LMT: Left main trunk
LAD: Left anterior descending artery
LCX: Left circumflex artery
IVUS: Intravascular ultrasound.

## Supplementary Materials

Supplementary 1 Video 1. Left coronary angiography (right anterior oblique caudal view) revealed a dissection at the proximal site of the LAD extending from the LMT, suggestive of type 1 spontaneous coronary artery dissection.

Supplementary 2 Video 2. The IVUS-guided rewiring technique was performed, and the third guide wire was inserted from the true lumen of the LCX to the true lumen of the LAD using a double-lumen catheter.

Supplementary 3 Video 3. The IVUS imaging after successful rewiring revealed that the third guide wire passed entirely through the true lumen from the LMT to LAD.
